# *Caretta caretta* nesting activity on Akumal Beaches, Mexico

**DOI:** 10.1038/s41598-020-60018-1

**Published:** 2020-02-20

**Authors:** J. M. González, R. Anastácio, H. A. Lizárraga-Cubedo, M. J. Pereira

**Affiliations:** 1 Centro Ecológico de Akumal, Quintana Roo, Mexico; 2 AFPR-BB, Aveiro, Portugal; 3 Departamento de Biologia e CESAM, Universidade de Aveiro, Aveiro, Portugal

**Keywords:** Behavioural ecology, Animal behaviour

## Abstract

Mexico has made substantial contributions to marine turtle protection and conservation, especially since 1990. Several conservation projects entail monitoring efforts to recover nesting territories for marine turtles. The Sea Turtle Protection Program of Akumal, in the Mexican state of Quintana Roo, was created in 1993 and was developed by the Akumal Ecological Center. This paper provides the nesting ecology parameters for *Caretta caretta* over a protection period of 24 years (1995–2018). A well-defined nesting peak was observed in June, with a nesting success rate of 75.2 ± 23.0%. Nesting females showed a mean curved carapace length of 99.0 ± 5.6  cm. The mean clutch size was 108.6 ± 24.6 eggs, with variation among years. The mean incubation period was 57.2 ± 6.2 days. The hatching and emergence success rates were 87.2 ± 16.9% and 78.8 ± 24.4%, respectively. For the 926 tagged females that returned, the remigration interval peaked at 726 days, with a 12-day inter-nesting period. The results show not only the recovery of the nesting population over time but also a decrease in female size; we postulate that this decrease is due to the recruitment of young females, which has been increasingly pronounced since 2010. Hence, the Akumal rookery plays an important role in its corresponding regional management unit (Atlantic Northwest).

## Introduction

The Quintana Roo beaches of the Yucatán Peninsula belong to the Western Caribbean Marine Ecoregion (the scale unit in the Marine Ecoregions of the World system) in the tropical northwestern Atlantic^1–3^. The beaches in this ecoregion served as nesting^4^ and foraging grounds^5,6^ for marine turtles long before humans settled on the American continent^7^. Human pressures have impeded sea turtle habitats in many ways. Today, tourism and recreational activities in these areas constitute threats^5,8,9^ to the recovery of small but important rookeries^10^. However, the conservation of nesting grounds has also flourished, which has successfully enabled marine turtles to nest on protected beaches^11^.

In the case of loggerheads, the “available long-term series of annual nest counts shows an overall increase over the past three generations for the northwest Atlantic loggerhead subpopulation, which breeds mostly in the southeastern U.S. and the Yucatán Peninsula in Mexico”^11^. Hence, loggerheads belong to the “least concern” category of the International Union for the Conservation of Nature (IUCN) Red List in the subpopulation/regional management unit (RMU) of the Atlantic Northwest^11^.

The geographic distribution of subgroups in the area is complex: five subpopulations of loggerheads have been identified; the “Greater Caribbean subpopulation”, which includes Akumal loggerheads, is one of these subpopulations^12^. This subpopulation is also considered one of the five recovery units within the northwest Atlantic^11^. In this context, there is evidence of loggerhead metapopulations^13^, which justifies the designation of a RMU (Atlantic Northwest RMU for loggerheads)^3^. Trends show that nesting has increased in several rookeries, aided by conservation projects (*vide*^11,14,15^).

**Figure 1:**
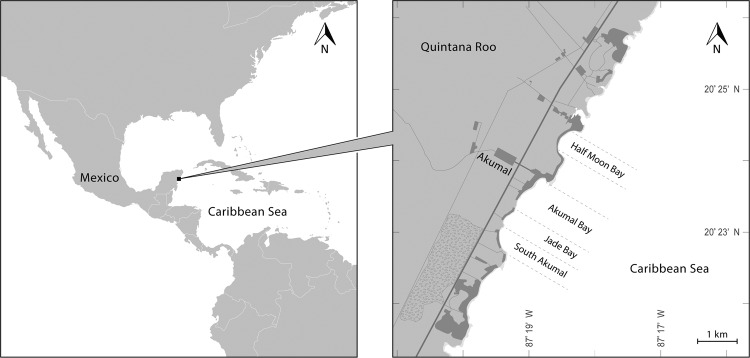
Study beaches at Akumal, Quintana Roo state, Mexico: Half Moon Bay (HMB), Akumal Bay (AB), Jade Bay (or Playa Tortugas (PT)), and South Akumal Bay (SA). The image on the left was designed by Freepik (https://www.freepik.com/free-photos-vectors/travel Travel vector created by Layerace - www.freepik.com) and modified using Adobe Illustrator CC2017; the right image was designed with Adobe Illustrator CC2017 from Landsat images (Landsat image courtesy of the U.S. Geological Survey).

The Akumal Ecological Center (CEA) Conservation and Research Program was founded in 1995 and has since been protecting marine turtle nesting beaches and foraging grounds (Akumal Bay, Fig. 1). For 24 years, conservation efforts have entailed collecting data, helping restore nesting sites and protecting nesting females and hatchlings of two species: *Chelonia mydas* and *Caretta caretta* (Cc). We present data from the CEA monitoring programme from 1995–2018 concerning nesting parameters, hatching success, and temporal distributions of loggerhead (Cc) sea turtle nests. We also debate the implications of the programme and provide recommendations for the future.

## Results

### Records

**Figure 2:**
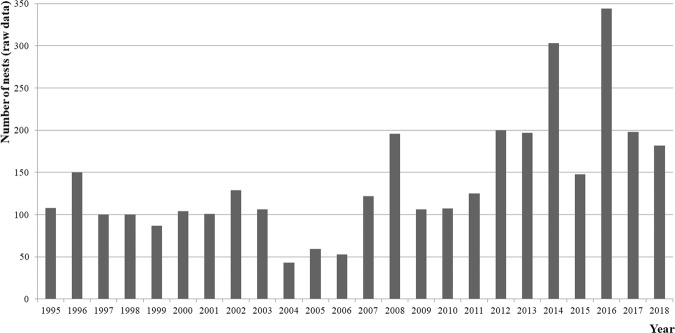
Total number of Cc nests between 1995 and 2018 recorded by CEA, Akumal.

**Figure 3:**
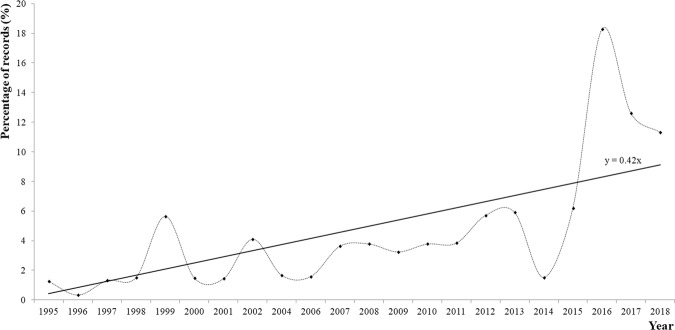
Percentage of total records obtained from beach monitoring of nesting Cc females between 1995 and 2018.

**Figure 4:**
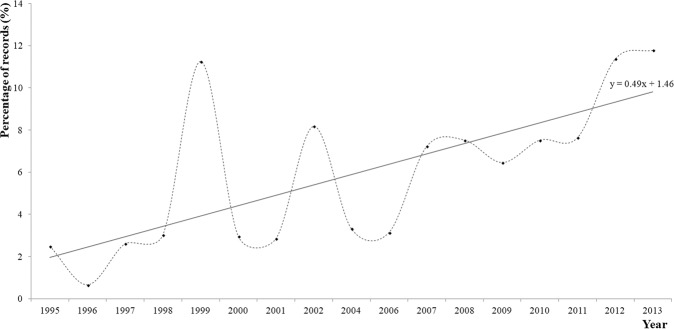
Percentage of total records obtained from beach monitoring of nesting Cc females between 1995 and 2013.

The raw CEA records of the total number of nests per year between 1995 and 2018 are shown in Fig. 2. For the statistical analysis, a total of 3364 (N) records were analysed. Figures 3 and 4 show the distribution of records per monitoring year as percentages. An increasing tendency is shown by the linear tendency lines drawn from the percentages of records of Cc that emerged during the project (Figs. 3 and 4).

Figure 3 shows that the number of records has peaked since 2015, *i.e*., an increased number of records was observed on Akumal beaches in the last four years of the project.

The last peak (2016) was different from the peaks in 1999, 2002, 2007/08 and 2012/13; additionally, the records obtained between 1995 and 2013 represented only 50.1% of all records analysed in this study.

### Morphometric analysis

**Figure 5:**
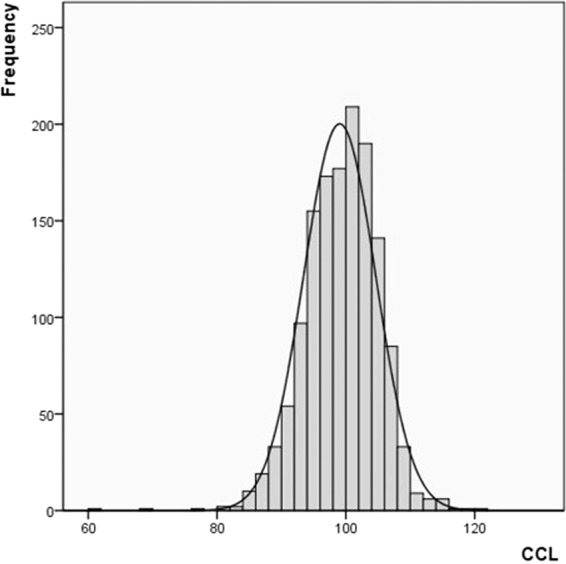
Histogram of CCL records of Cc measured during the Akumal Project (1995–2018). The first bars are for 3 turtles that deposited nests; these individuals were small and probably new recruits.

For the nesting Cc turtles, the mean curved carapace length (CCL ± SD) was 99.0 ± 5.6 cm (N = 1407 females measured), ranging from 61.1–120.0 cm (Me = 99.3 cm and Mo = 100.0 cm). The histogram in Fig. 5 shows the frequency distribution for the CCL of all samples.

The average Cc curved carapace width (CCW) was 90.5 ± 5.7 cm (N = 1406), ranging from 59.0–111.0 cm (Me = 91.0 cm; Mo = 90.0 cm).

The 24 years of carapace measurements show a Gaussian distribution. The ANOVA for CCL showed significant differences between 1995 and 2015; between 1998 and 2012, 2015, and 2018; between 1999 and 2012, 2015, and 2018; and between 2000 and 2012, 2015 and 2018 (F = 3.795; P = 0.000).

The trio of 1998/1999/2000 showed the highest average CCLs (approximately 101 cm), and the values during these years differed significantly from those in 2012/2015/2018, when the smallest averages were recorded (approximately 97 cm). Similar differences in clutch sizes were also expected to be detected in these years. In fact, the nesting seasons of 2015 and 2018 showed the smallest clutch sizes and contrasted significantly (F = 3.385; P = 0.000) with the years with the largest clutch sizes (1999, 2007, 2008, 2011, and 2017).

**Figure 6:**
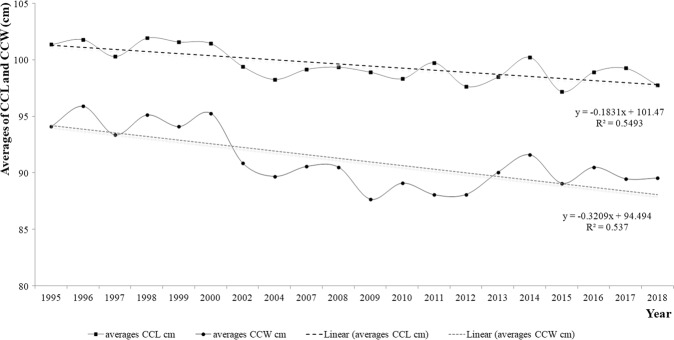
Variation in CCL and CCW averages of emerged Cc sea turtles between 1995 and 2018 on Akumal beaches.

Additionally, the variation in morphometrics decreased during the study period (Fig. 6).

### Clutch size and nest parameters

**Figure 7:**
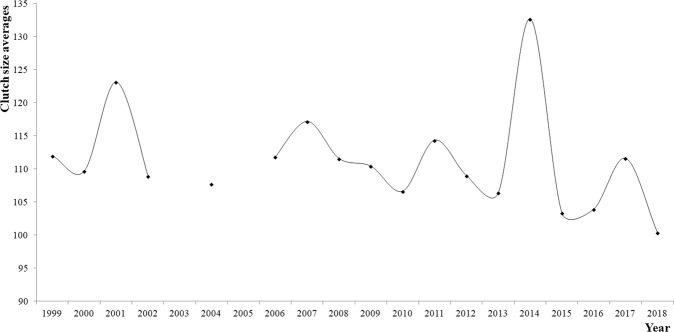
Clutch size averages for Cc in Akumal over the years.

The nesting success for Cc was 75.2 ± 23.0%, and the average clutch size was 108.6 ± 24.6 eggs (N = 1710 nests). There were no data regarding the nest contents in 2003 or 2005 (Fig. 7). The year 2018 showed the smallest average clutch size (100.4 ± 23.0 eggs), in contrast with 2014, which had the highest average (132.7 ± 24.9 eggs). The number of clutches in 2014 was atypical since the field effort was compromised (there were no nesting parameter data). The year with the highest average incubation period (IP) value (2010) also had a small clutch size average.

**Figure 8:**
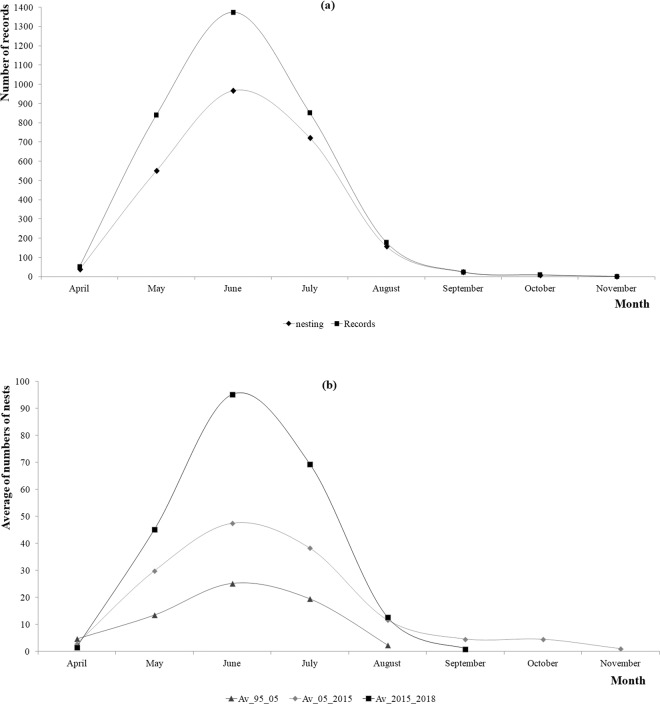
(**a**) Turtle activity per month recorded *versus* number of records during the same months. The “nesting” line is also the number of nests.month^−1^ from 1995 until 2018. (**b**) Nesting season averages over a 10-year period for Akumal (1995–2005; 2005–2015; 2015–2018).

The nesting season is shown in Fig. 8a; it occurs between April and November, but most nesting activity occurred between May and July. June is the peak nesting month for Cc in Akumal. There was no disruption of the nesting season over the 24 years (Fig. 8b). The last years of the programme showed increases in nests.month^−1^ in June and July (more than 25%; Fig. 8b).

### Incubation period (IP)

**Figure 9:**
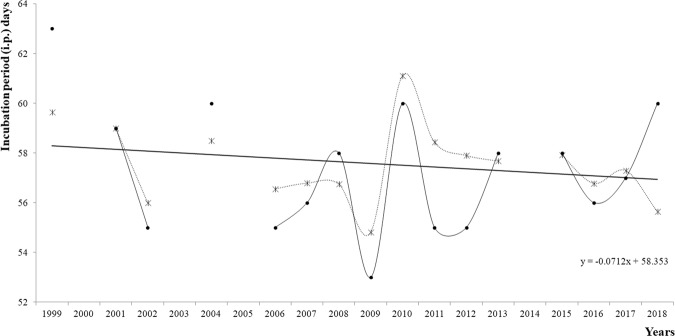
IP values per year: dots represent the mode values for the IP; asterisks represent the IP averages. The data needed to determine the IP averages were lacking during four years (*i.e*., 2002, 2003, 2005, and 2014).

The overall (1995–2004) average IP was 57.2 ± 6.2 days (Table 1), ranging from 39 to 81 days (Mo = 55.0 days; Me = 57.0 days; N = 1413). The IP showed a slight decreasing tendency over the years (Fig. 9), achieving its lowest peak in 2009 (IP = 54.8 ± 4.8 days) and its highest peak in 2010 (IP = 61.1 ± 6.7 days).

**Table 1:** Mean results of the data for nesting females (1995–2018).

Parameters	Incubation period (days)	Clutch size (number of eggs)	Hatching success (%)	Emergence success (%)
N	1413	1710	1646	823
Mean ± SD	57.2 ± 6.2	108.6 ± 24.6	87.2 ± 16.9	78.8 ± 24.4
Median	57.0	109.0	92.8	88.0
Mode	55.0	106.0	100.0	0.0

### Hatching success (HS) and emergence success (ES)

Generally, HS and ES rates are high in a successful conservation programme. For Cc, the overall HS was 87.2 ± 16.9%. The ES was 78.8 ± 24.4%, with much variability (in fact, the values ranged from 0–100%, meaning that in some of the excavated nests, all the hatchlings (alive or not) were found in the sand). Table 1 summarizes the overall IP, HS and ES averages for Cc, and Table 2 shows the overall averages for each nest parameter.

**Table 2:** Averages for the categories of Cc nest contents on Akumal beaches.

Parameters	Emerged	Dead in nest	Live in nest	UH	UHT	Predated
N (number of nests sampled)	822	1534	1581	1536	773	423
Mean ± SD	84.3 ± 33.3	1.8 ± 5.7	53.1 ± 50.4	11.4 ± 16.5	5.2 ± 10.3	1.3 ± 5.6
Std. Error of the Mean	1.2	0.1	1.3	0.4	0.4	0.3
Minimum	0	0	0	0	0	0
Maximum	205	85	170	144	94	52

### Inter-nesting period and remigration interval

**Figure 10:**
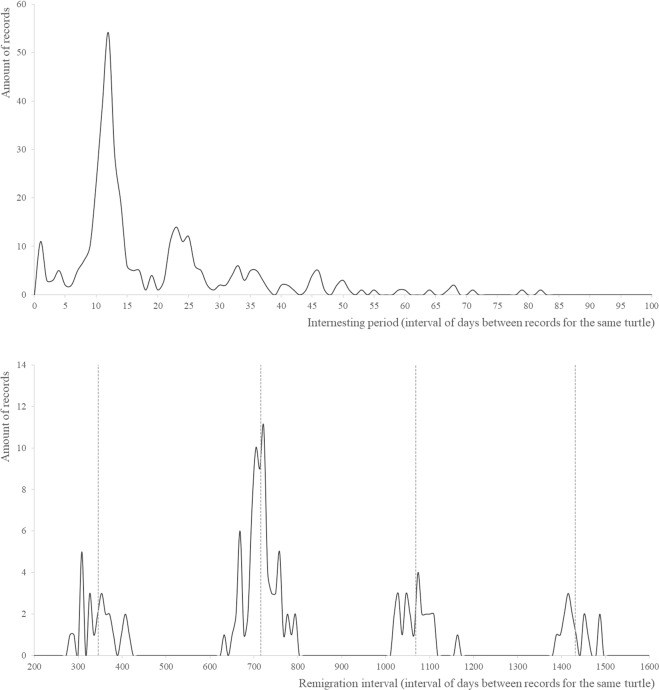
Upper chart: inter-nesting period for Akumal tagged females. Lower chart: remigration interval plot. The dashed line: average of the values for the cluster of corresponding values around it.

Tag information revealed that for the Akumal beaches, Cc females returned 12 days after the first emergence (Fig. 10; first chart). The remigration interval was 726.0 days (1.99 years) for 926 unique tags (N). Data dispersion was high (Fig. 10; second chart); the mean of the first cluster of data (Fig. 10b) was approximately 346.1 days, the mean of the second cluster of data was 715.4 days, and the mean of the third cluster of data was 1068.2 days. The averages of the four clusters (Fig. 10b) showed an effective Cc remigration cycle of exactly 345.4 days between the four clusters.

### Effects on nesting on each beach

**Figure 11:**
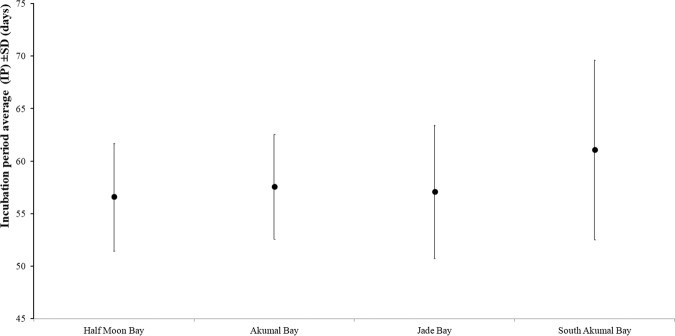
Average IP for the four main beaches included in the project.

Incubation occurred most frequently in PT and HMB (Fig. 11). However, the clutches were largest in SA, which has the fewest disturbances attributable to tourists and lights from hotels and houses. AB, for example, is very exposed to tourism since two major hotel complexes are present on this beach. HMB also has many houses and tourists. The IP also differed among these beaches. The overall IP average was the longest in SA and the shortest in HMB.

ANOVA by beach suggested that the IP in SA was significantly different from the IPs on the other beaches (P < 0.006; Tukey post hoc test).

**Figure 12:**
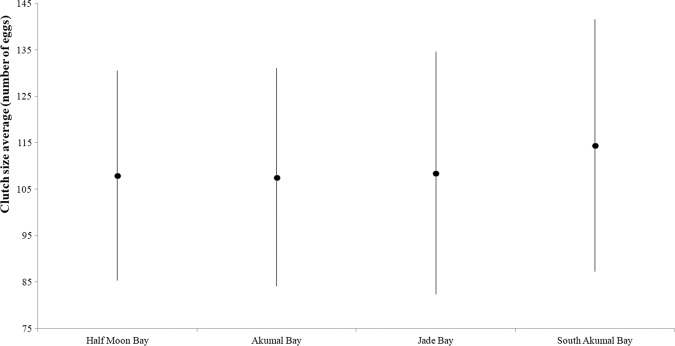
Clutch size averages for the four main beaches included in the project.

Although the average clutch size in SA was higher than those on the other three beaches, there were no significant differences between them (Fig. 12).

The HS rates in HMB and PT were significantly different (P < 0.001; Tukey’s post hoc test). Additionally, the ES significantly differed between PT and AB (P = 0.003), HMB (P < 0.001), and SA (P = 0.025) according to Tukey’s post hoc tests.

## Discussion

### What do the numbers show

The Cc females that were observed in Akumal were increasingly frequent but decreased in size in the last years of the conservation programme. It is probable that many Cc born on Akumal beaches were recruited to nest, showing philopatric behaviour.

The results show that the Cc nesting in Akumal deposited an increasing number of nests, especially since 2010. Although Mexico’s Protected Area Commission (CONAMP)^16^ indicated a 5% decline in nesting for Cc between 1995 and 2006 based on the analysis of records from index beaches (*i.e*., beaches that provide data to estimate the trends for the region), the IUCN reported that data from the Greater Caribbean Recovery Units suggested the opposite^11^. The Akumal Project corroborated IUCN’s results but showed that turtles preferred the beaches in Akumal for other reasons.

The increase in nesting numbers in Akumal warrants explanation. Marine turtle conservation projects have been increasing in the Caribbean, and accordingly, so have the management skills of the monitors^16^. Mexico fully prohibited the capture of marine turtles in its waters in 1990^16,17^. As more turtles were protected, more turtles were recruited to the waters and nesting grounds as they reached sexual maturity. The marine turtles that were protected in 1995 in Akumal would reach this maturity after 18/20 years (2013/2015; *vide* the following section), and in fact, 50% of the analysed data were concentrated after 2013 (the last five years of the project). The monitors working for more than 20 years in the area who marked the marine turtles recorded successful nesting by the same turtles on Akumal beaches approximately 18 years later. This view is also corroborated by Labrada-Martagón *et al*.^6^.

### Morphometry of nesting females

Chaloupka and Limpus^18^ studied and defined “age classes” for loggerhead turtles. An adult CCL ranges between 85 and 105 cm. The CCL average in Akumal indicated that the nesting females were adults, though a few small-sized females nested on its beaches. The decreasing average CCL value over the years (Fig. 6) was also observed in other scientific studies; “Karen Bjorndal revealed that somatic growth rates for loggerheads (…) throughout the region began to decline in the late 1990s as the result of an ecological regime shift; the decline continues to the present”^19^. What does this ecological shift entail? Does increased sea surface temperature accelerate growth and sexual maturity? Will relatively small females with a fast growth rate be observed? Or do the numbers mean that the number of young females in the population increased in the last several years? An increased rate of recruitment for first-time breeders may explain the increase in the population registered in recent years.

### Nesting parameters

Nesting success in Akumal was higher than that reported in Guanahacabibes Peninsula, Cuba (67%), which belongs to the same RMU. Nesting success values are probably affected by the tourism pressure on three of the main nesting beaches. Light pollution, obstacles in the sand concentrated in specific areas (such as beach furniture), and people on the beach can lead Cc turtles to abandon their nesting attempts. In some areas of the beach, the sand may be too dry or too thick to excavate, with rocks and coral debris, which also leads turtles to abort their nesting intentions.

The remigration interval was 2.0 years, which is in accordance with other publications (*vide* Hart *et al*.^12^), and Cc takes 12 days on average to re-nest/-emerge in the same nesting season. The analysis showed a very predictable population of nesters exhibiting clockwork-like nesting behaviour. For these turtles, nesting seasons occur precisely every 354.4 days on average. Another aspect revealed by the tag analysis was that the first emergence in a nesting season occurred on the exact same beach as it did in the previous nesting season.

The typical average clutch size for Cc is 100–130 eggs^20^, meaning that the average clutch size in Akumal (108.6 ± 24.6 eggs) exhibits a large variation. In Florida, the average clutch size for Cc is 98.5 ± 1.7 eggs^21^, which is smaller than that in Akumal, and in the Archie Carr National Wildlife Refuge along the central coast of Florida, the average clutch size is 113.9 ± 1.4 eggs according to Ehrhart *et al*.^14^, which is higher than that in Akumal. High clutch sizes were observed in 2014, but there were no significant differences between the average CCL in this year and the average CCLs in other years in the study period. Hence, the difference in clutch size may be due to causes other than female dimensions, even though there were no significant differences between the clutch size in 2014 and the sizes in other years. These turtles are not particularly large (CCL = 100.2 ± 4.9 cm), but they lay a large number of eggs per clutch.

The average IP for Cc in Akumal was longer (57.2 ± 6.2 days) than the published value of 50.8 ± 1.2^22^, but it was within the range of other studies (46 to 82 days for Matsuzawa *et al*.^23^ study). The range of values was high, probably because there was high seasonal variation in the temperature of the sand.

Since the pivotal IP is approximately 52.6 days in the Mediterranean^24^, one can hypothesize that a balanced ratio of males and females per nest is produced in Akumal. Additionally, the IP depends on temperature fluctuations^25^. The pivotal temperature for Cc incubation is 28.74 °C^26^, although Mrosovsky *et al*.^24^ determined this value to be 29.3 °C. Another recent experimental study emphasized that the optimal range for Cc incubation was 28.5–31 °C^25^. Temperatures above 31 °C may impact the hatchling survivorship rate^25^, which probably explains why some ES values were so low. Humidity, air temperature, and precipitation are probably the main climatic drivers of hatchling production; sea surface temperature and wind speed, though important, do not have significant influences^27^. It would be very important to determine, for example, the temperature fluctuations in Akumal sand/nests to understand how they affect incubation conditions (are they female-biased with an increasing trend?).

The Cc HS values were similar or even increased when compared to those in other studies (*e.g*., similar to 87.3 ± 17.8%^27^; higher than 68% ± 4%^21^, 55.1 ± 4.0%^14^), although the ES was decreased and varied most likely due to the difficulties faced by hatchling when leaving the nest. Abiotic factors in Akumal vary due to strong precipitation or flooding due to storms and hurricanes and cause pre-emergent mortality^23^. In a Florida study, the HS rates for loggerheads decreased from 1985–2003^21^. In a study by Ehrhart *et al*.^14^, the HS and ES values were very low due egg washing caused by beach erosion (55.1 ± 4.0%; 53.3 ± 3.7%). On Japanese beaches, the HS determined by Matsuzawa *et al*.^23^ was relatively low, which was possibly due to the following conditions: compacted/desiccated sand, hatchlings trapped in the nest due to heat (inhibition of movement), or oxygen deficiency inside the nest due to accelerated metabolic rates^23^. It is possible that in Akumal, the ES is compromised by one of these constraints; this possibility needs to be further considered.

### Conservation implications

Akumal, where snorkelling and observation of nesting females are possible activities for tourists, is certainly important in many aspects^8^. These opportunities have provided alternative livelihoods for villagers that settled in the region, a pattern observed in other southeastern Mexican coastal locations (*vide* the Kanzul beach case^9^) and in other Caribbean locations^5^.

On the beaches and in the foraging grounds off the southeastern Mexican coast, efforts have been made to enhance the protection of juveniles, females and nests. Activities related to tourism are more efficiently controlled by local environmental authorities^28^. Additionally, it is very important to improve citizens’ awareness of the recovery and protection of nesting, development and foraging territories. For example, touristic developments should focus on offering information and responsible activities to tourists. The respect of sea turtle habitats and niches by people is crucial. *Cc* is still vulnerable even with all the apparent recovery suggested by the numbers and indicators and the protection provided by the conservation teams.

To guarantee the success of the *Inter-American Convention for the Protection and Conservation of Sea Turtles* agenda in the future, other measures need to be taken. Cases such as those in the Cayman Islands, where migratory green and loggerhead nesting populations are critically reduced^13^, must be addressed. The protection of all territories and the interconnections among them inside the RMU will provide additional opportunities for population recovery. Additional evidence, such as the results obtained by Blumenthal *et al*.^29^, who emphasized that “oceanic juveniles from some rookeries appear to be dispersed among multiple foraging grounds, while those from other rookeries appear to be more locally constrained”, must also be considered to maximize protection.

Efforts are needed to identify the role of the rookeries in Akumal and other Quintana Roo regions in supporting the migratory subpopulations/cohorts of the RMU (*e.g*., for the Florida Atlantic coast, the Gulf of Mexico, the Cuban and Bahamian waters, and even the eastern Atlantic waters^10,30–32^, among other destinations). Field biologists are collecting data and filling gaps to enhance the knowledge of these long-lived species^19^. The information provided here provides indicators for the Yucatán Peninsula and can be used to compare nesting parameters with other rookeries inside the RMU. Genetic^30^ or telemetric studies and cross-tagging information analyses are mandatory. These approaches can help reveal the potential connections, genetic drift of genes, molecular diversity and the metapopulation^29^ structure within the wider Caribbean region. The State of the World’s Sea Turtles (SWOT) has emphasized the need for cooperation among teams.

## Materials and Methods

### Monitoring programme and field effort

In Mexico, this species is protected under Federal laws and specific Norms such as NOM-059-SEMARNAT-2010^33^, which protects threatened species of flora and fauna, and NOM-162-SEMARNAT-2012^34^, which specifies the protection, recovery and management of the sea turtle populations and their nesting habitats. In Akumal, the nesting season is from May to November/December^4,34,35^. Four main beaches are protected by the Akumal Ecological Center (Fig. 1) and monitored daily during the nesting season. Every night during the nesting season, the beaches were patrolled from 9 pm until 4 am and every morning (6 am) when eggs began to hatch by instructed monitors. During these patrols, several variables were observed/collected on paper forms, and detected nests were identified with a tag (*e.g*., BA Cc 0001). The nest variables were also collected. After the IP and according to NOM-162-SEMARNAT-2012^34^ protocols, each nest was excavated, and the shells, dead hatchlings, and live hatchlings were counted.

Nesting sea turtles were tagged following the Eckert and Beggs^36^ methodology with Monel tags, which were generally applied between the 2^nd^ and 3^rd^ plates on one of the front flippers. Prior to tagging, marking scars and other tags were checked on both the flippers and paddles. Nests were carefully monitored after the first evidence of hatching; they were not disturbed until there was evidence of the emergence of hatchlings. Many emergence events were observed *in situ* since the beaches are on small bays. Three to five days after the eggs hatched, the nests were excavated to perform counts.

### Collected/measured variables

For each patrol time, the date (and sometimes the GPS coordinates) of the emergence/nesting events, curved carapace length (CCL, notch to tip, n-t) and width (CCW) were recorded according to Bolton’s method^37^, and the track width, presence of tags and respective codes, and presence of epibionts on the carapace were among the variables collected during the night patrols. Whenever a nest was constructed, an identification code was attributed; if oviposition was observed, variables such as nest depth and temperatures inside the nest and of the sand were collected. Additionally, whenever possible, the number of eggs was counted. After incubation, the following nest content variables were recorded: emerged hatchlings, shells, live and dead hatchlings, undeveloped eggs, unhatched and unhatched term (UHT) (“unhatched apparently full-term embryo in eggshell or pipped, *i.e*., with a small amount of external yolk material”^38^) eggs, and predated eggs. Nests were also monitored during incubation, and relevant events were recorded (flooding, predation events). All nests at risk after oviposition were moved to the hatchery in Jade Bay (Fig. 1). The computed variables were the IP, clutch size, hatching success (HS) and emergence success (ES), inter-nesting interval and remigration interval.

### Study site/beaches

The CEA patrols and protects four main beaches: Half Moon Bay (HMB), Akumal Bay (AB), Jade Bay (or Playa Tortugas (PT)), and South Akumal Bay (SA) (Fig. 1). The project has a hatchery situated in PT. The four beaches are composed of biogenic sand, and PT hosts the largest number of nests.

Each monitored beach has geographical, biological and physical differences, as well as different lengths and widths. HMB is 700 m long and 18 m wide at the widest part and 5 m wide at the narrowest part. The beachfront contains approximately 80% human development, such as condo buildings and rental houses. AB is approximately 1200 m long; however, only 900 m serves as a nesting area due to sea turtle preference. Approximately 90% of the beachfront is occupied by hotels and restaurants. The width varies from 5 to 20 m. PT is 500 m long, with 60% human occupancy by one low-density hotel, one beach club and houses. Due to these structures, the minimum width is 2 m at some sites and 10 m at the widest part. SA has the smallest nesting area, at approximately 400 m long; however, the total length of the beach is 700 m. Due to the beach’s shape, the central area is 20 m wide and has the highest density, and the ends are only 2 m wide. This beach has the least human disturbance, hosting only private houses^39^.

### Statistical analysis

The statistical analysis was performed with PASW Statistics 18, from SPSS an IBM company, and Microsoft Office Excel 2007 software. Significance was estimated at the 95% confidence level. Variables such as IP, clutch size, HS and ES were compared using t-tests and one-way ANOVA and Games-Howell, Tukey’s or Scheffe’s post hoc tests when statistically significant differences were detected (p < 0.05). For all the variables, the overall data were estimated, and then averages for each nesting season (which in this case coincided with the annual data) were determined; the annual values were used for ANOVAs.

To determine the inter-nesting period, the remigration interval tag numbers of the females that emerged were used. The inter-nesting interval was considered the number of days between a successful nesting event and the first subsequent attempt by the same female in a particular nesting season; the remigration interval was considered the period between the observations of a female in different but subsequent nesting seasons^20^. The analysis was complex due to the number of tags and records. Therefore, an automation process in Microsoft Office Excel 2007 was required to run the data and generate the two variables. The automation formulas consisted of identifying the unique tag numbers in the overall sample (all years), the number of times a unique tag was repeated, and the date of each repetition; building a matrix for the tags; and finally calculating the difference (number of days) between the dates.

### Ethical approval and informed consent

Fieldwork in Akumal, Mexico, was conducted by the staff from the non-governmental organization Centro Ecológico de Akumal (CEA) in accordance with the guidelines and permits (non-extractive use of sea turtle permits, with the following references SGPA/DGVS/04368/17, SGPA/DGVS/006855/18) provided by SEMARNAT, Secretaria de Medio Ambiente y Recursos Naturales, and through the Dirección General de Vida Silvestre (DGVS). All methods were carried out according to Mexican regulations (*vide* NOM-059-SEMARNAT-2010^33^, NOM-162-SEMARNAT-2012^34^) imposed by CEA and SEMARNAT. All experimental protocols were approved by CEA and SEMARNAT.
